# Prognostic role of FDG-PET in patients with relapsed/refractory large B-cell lymphoma treated with CD3-CD20 directed bispecific antibodies: a multicentric analysis

**DOI:** 10.1007/s00259-026-07958-4

**Published:** 2026-06-01

**Authors:** Sophie Wollnitza, David Ventura, Marcel Teichert, Fabian Freisleben, Hristo Boyadzhiev, Robert Seifert, Michelle Amon, Francesco Barbato, Julia Katharina Scholz, Michael Beck, Andrea Kerkhoff, Akhil Behringer, Philipp Backhaus, Alexander Pohlmann, Philipp Schindler, Wolfgang Roll, Fabian Müller, Urban Novak, Thomas Pabst, Michael Schäfers, Bastian von Tresckow, Evgenii Shumilov, Georg Lenz

**Affiliations:** 1 Department of Medicine A, Hematology, Oncology and Pneumology, University Hospital Muenster, Muenster, Germany; 2 Department of Nuclear Medicine, University Hospital Muenster, Muenster, Germany; 3 European Institute for Molecular Imaging (EIMI), University of Muenster, Muenster, Germany; 4 Department of Hematology and Stem Cell Transplantation, West German Cancer Center, German Cancer consortium (DKTK partner site Essen), University Hospital Essen, University of Duisburg-Essen, Essen, Germany; 5 Department of Nuclear Medicine, University Hospital Essen, Essen, Germany; 6 Department of Medical Oncology, University Hospital Bern, University of Bern, Bern, Switzerland; 7 Department of Nuclear Medicine, University Hospital Bern, Bern, Switzerland; 8 Department of Internal Medicine 5, Hematology and Oncology, University Hospital of Erlangen, Friedrich-Alexander-Universität-Erlangen Nürnberg, Erlangen, Germany; 9 Department of Nuclear Medicine, University Hospital of Erlangen, Friedrich-Alexander- Universität-Erlangen Nürnberg, Erlangen, Germany; 10 Department of Radiology, University Hospital Muenster, Muenster, Germany

**Keywords:** R/r LBCL; BsAb, FDG-PET, Deauville-Score

## Abstract

**Purpose:**

The clinical use of CD3–CD20–directed bispecific antibodies (BsAbs) has significantly improved outcomes of patients with relapsed/refractory LBCL. However, up to 50% of patients relapse. This study evaluated the prognostic value of longitudinal FDG-PET parameters.

**Methods:**

58 patients with baseline PET and 45 (78%) with first PET after BsAb initiation were included. Standard PET metrics and Deauville score (DS) were recorded, with percent changes at follow-up defined as ΔPET parameters. Prognostic impact of DS and semi-quantitative PET metrics were determined for PFS/OS.

**Results:**

At baseline an whole-body MTV > 504.7 cm³ was associated with shorter median PFS of 1 vs. 3 months (HR = 5.6, *P* = 0.00025; C-index = 0.94). At first follow-up, those with DS4–5 exhibited significantly higher risk of progression (HR = 11.42, *P* = 0.0182; C-index = 0.97) and shorter survival (HR = 7.07, *P* = 0.059; C-index = 0.72). Non-achievement of ≥ 75.5% SUVmean reduction significantly increased risk for PFS (HR = 7.65; *p* = 0.0065; C-index = 0.92) and OS events (HR = 11.1; *p* = 0.001; C-index = 0.95).

**Conclusion:**

In summary, longitudinal semi-quantitative PET assessment identified patients at high risk of relapse and may enhance risk stratification and personalized management in BsAb-treated patients.

**Supplementary Information:**

The online version contains supplementary material available at 10.1007/s00259-026-07958-4.

## Introduction

Relapsed or refractory large B-cell lymphoma (r/r LBCL) continues to represent a major therapeutic challenge [1]. First-line R-CHOP-like (rituximab, cyclophosphamide, doxorubicin, vincristine and prednisone) immunochemotherapy regimes cure approximately 60–70% of all patients, still leaving a considerable proportion with refractory disease or early relapse, both being associated with a poor prognosis [2, 3].

Over the past few years, the introduction of CD3-CD20 directed bispecific antibodies (BsAbs) has significantly changed the therapeutic armamentarium and also prognosis of patients with r/r LBCL [4]. Currently, BsAbs are approved either as monotherapy after two prior systemic therapies or in combination with chemotherapy in first relapse for patients with r/r LBCL being ineligible for high-dose chemotherapy and autologous stem cell transplantation [5–7]. In the approval trials, monotherapies with BsAbs achieved complete remission (CR) rates of roughly 40%, with recent long-term follow-up data demonstrating durable remissions among CR responders suggesting a cure of the disease [5–8]. However, approximately half of the patients do not respond to treatment with BsAbs or relapse subsequently, and real-world analyses indicate lower CR rates compared with those reported in controlled clinical trial settings [9, 10]. Various clinical risk factors, including elevated lactate dehydrogenase (LDH), bulky disease, inflammation and/or early relapse following chimeric antigen receptor T-cell therapy (CAR-T) are consistently associated with inferior outcomes [11–13]. These observations underscore the necessity for reliable baseline and early on-treatment biomarkers to refine patient selection, guide treatment decisions, and identify individuals who may benefit from early treatment intensification or consolidation strategies.

[¹⁸F]fluorodeoxyglucose positron-emission-tomography (FDG-PET) is a well-established functional imaging tool routinely employed for staging, response assessment, and prognostication in patients with aggressive lymphomas. In patients treated with conventional immunochemotherapy and CAR-T, both baseline and early first PET parameters have shown strong prognostic relevance [14–17]. However, the prognostic value of FDG-PET during BsAb treatment remains insufficiently defined [5, 18]. Specifically, it remains uncertain whether baseline metabolic tumor burden or early metabolic response, as reflected by semi-quantitative PET parameters, can reliably predict treatment outcomes or provide individualized therapeutic strategies. Therefore, a multicenter analysis of German and Swiss patients was conducted aiming to evaluate the prognostic impact of baseline and first follow-up FDG-PET in patients with r/r LBCL undergoing BsAb treatment.

## Materials and methods

### Study design

This retrospective multicentric analysis was conducted by four university centers: Münster, Essen, Erlangen (Germany) and Bern (Switzerland). This study aimed to examine the prognostic value of baseline and first FDG-PET after initiation of therapy in patients that underwent treatment with BsAbs. The analysis was performed to evaluate the ability of FDG-PET to predict progression-free survival (PFS) and overall survival (OS), thereby facilitating the identification of patients with persisting disease. A total of 58 patients with r/r LBCL were included in this analysis. The FDG-PET scans were taken before treatment initiation (baseline PET) and at first follow-up after treatment initation. Ethical approval for this analysis was obtained from the Ethics Committee of Westphalia-Lippe and the Medical Faculty of the University of Münster (approval no. [2025-314-f-S]).

### Patients

The present study comprised patients diagnosed with r/r LBCL who had undergone BsAbs treatment and received FDG-PET prior to BsAb initiation and first follow-up. 58 patients were included in this multicenter analysis. The clinical and imaging data was retrieved from the medical records, electronic patient files, picture archiving and communication system, and hospital database, and were supplemented by patient-related documents. The patient data were locally pseudonymized, and summary statistics and end-point analyses were performed centrally.

### FDG-PET

PET scans were performed using a Siemens Biograph 128 mCT (Münster, Essen), a Siemens Biograph 64 Vision 600 (Essen, Erlangen), a Siemens Biograph Vision Quadra (Bern). After a minimum fasting period of 6 h and confirmation of blood glucose levels below 6.7 mM, patients received an intravenous injection of FDG at a dose of 3.5 MBq/kg body weight (Münster, Essen (Siemens Biograph 128 mCT in patients weighing < 100 kg)), 2.8 MBq/kg body weight (Essen (Siemens Biograph 128 mCT in patients weighing > 100 kg)), 3 MBq/kg body weight (Bern, Essen (Siemens Biograph64 Vision 600), Erlangen). PET data were acquired 60 ± 10 min after tracer administration, covering the region from the head to the mid-thigh. A low-dose CT scan was obtained for attenuation correction. In selected cases, an additional diagnostic CT scan was performed using a non-ionic iodinated contrast agent.

### Image analysis

**Fig. 1:**
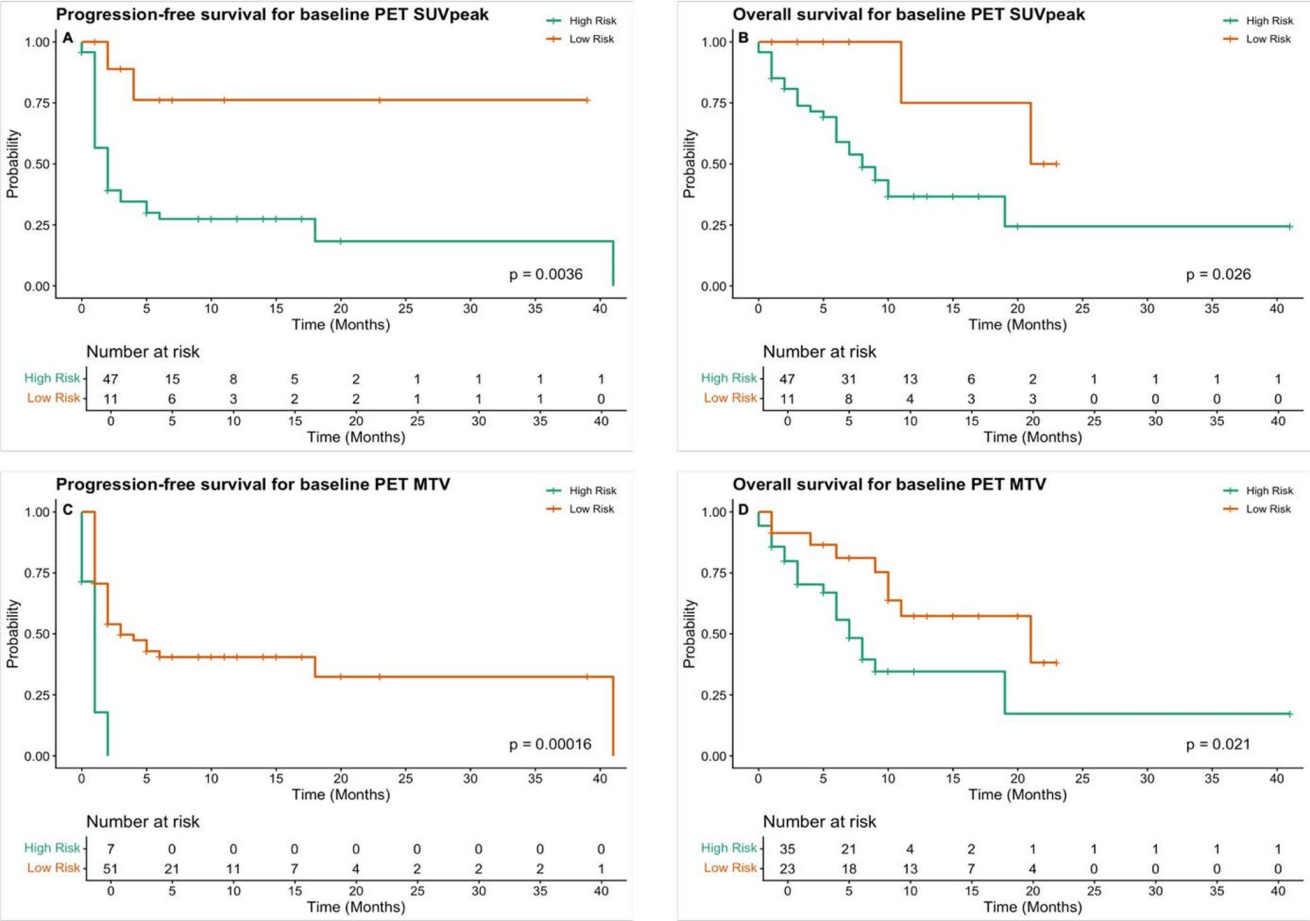
Kaplan-Meier analysis of binarized ideal cut-off PET parameters (SUVpeak, whole-body MTV) in the baseline PET. Patients are separated in a high-risk and low-risk group towards shorter PFS (**A**, **B**) and OS (**C**, **D**)

All PET scans were jointly evaluated by two experienced nuclear medicine physicians at each center. The total FDG-avid tumor volume was semi-automatically segmented using the Lesion Scout tool implemented in the imaging software syngo.via (Siemens Healthineers). For standardization in accordance with current guideline recommendations, PERCIST threshold (1.5 × liver SUVmean + 2 SD) was used to identify elevated FDG-positive areas, followed by SUVmax-based volumetric segmentation using a 41% threshold of each individual selected lesion [19, 20]. After semi-automatic segmentation, missing lesions were added and physiological or non–lesion-related uptake was manually excluded in consensus by the PET readers on a per-case basis. A relative SUVmax-based threshold was applied to account for heterogeneous FDG uptake patterns and to include follow-up PET scans. Standard PET parameters (SUVmax, SUVpeak, SUVmean, SUVmin) and Deauville score (DS) were derived from the lesion with the highest uptake [21]. Metabolic tumor volume (whole-body MTV, cm³) and total lesion glycolysis (whole-body TLG, calculated as whole-body MTV × SUVmean) were quantified using the aforementioned segmentation approach (Fig. 1S) [20, 22]. For first PET after BsAb initiation assessments, patients were considered to have achieved a complete metabolic response if no measurable whole-body MTV or whole-body TLG values were detected. In these cases, SUV parameters were derived using a 1 cm³ volume of interest (VOI) placed in the region of highest lesional FDG uptake, as defined at baseline. Changes in all PET-derived metrics were additionally evaluated as ΔPET parameters, calculated as the ratio of follow-up to baseline values. Deauville scores 1–3 (DS1–3) were classified as complete metabolic response (CMR), whereas Deauville scores 4–5 (DS4–5) were categorized as incomplete metabolic response [14, 21]. Patients with progression at first PET after BsAb initiation according to interdisciplinary tumor board decision were excluded from follow-up analysis.

### Statistical analysis

PET parameters are reported as absolute values, whereas ΔPET parameters are expressed as percentages. Clinical and demographic variables are presented as absolute numbers, percentages, ranges, and 95% confidence intervals (95% CI). Scanner-related variability of PET parameters was assessed separately for baseline, relative follow-up, and absolute follow-up datasets. Group differences across scanners were evaluated using Kruskal–Wallis testing with epsilon-squared (ε²) effect size estimation and Benjamini–Hochberg–adjusted pairwise Wilcoxon tests. In addition, log-transformed linear regression models including scanner as categorical predictor were performed to confirm robustness of the findings. Ideal cut-off values for PET and ΔPET parameters were determined using maximally selected log-rank statistics (MSLRS), with p-values adjusted through a 1000-fold conditional Monte Carlo procedure (Maxstat package, R, version 4.5.1) and receiver operating characteristics (ROC) with area under the curve (AUC) calculation for verification of the aforementioned ideal cut-offs (pROC package, R, version 4.5.1). Based on these cut-offs, two risk groups were defined. The two risk groups as well as patients stratified by DS 1–3 or DS 4–5 were evaluated for PFS and OS using Kaplan–Meier analysis and Cox proportional hazards regression, with hazard ratios (HRs) used for risk stratification (Survival and Survminer packages, R, version 4.5.1). Risk group classification was assigned according to the HR for PFS and OS events in the Cox model, with patients at increased risk of progression or death designated as high-risk, and all others as low-risk. Model discrimination for Cox regression models was assessed using Harrell’s concordance index (C-Index; Survcomp package, R, version 4.5.1), which quantifies the concordance between predicted and observed survival times (values range from 0.5 = no discrimination to 1.0 = perfect discrimination). A multivariable Cox proportional hazards model with an univariate and multivariate analysis was used to derive an individual risk score (linear predictor) for both PFS and OS. Analyses were performed in the complete-case cohort, if applicable. For clinical applicability and to reduce overfitting, patients were primarily dichotomized into low-risk and high-risk groups using a pre-specified median cut-off of the linear predictor. In addition, an exploratory cutoff was determined using maximally selected log-rank statistics (MSLRS). The null hypothesis was rejected when p-value < 0.05.

## Results

### Patients’ characteristics

Detailed patients’ characteristics are summarized in Table 1. The study cohort included 58 patients with a median age of 62 years (range: 27–81). Overall, 42 patients (72%) were diagnosed with diffuse large B-cell lymphoma and 16 (28%) had a high-grade B-cell lymphoma (HGBL). Prior to the initiation of treatment with BsAbs, patients had already received a median of three lines of therapy (range: 1–8). All patients received an anti-CD20/anthracycline-based regime as first-line treatment and 76% had undergone platinum-containing immunochemotherapy following r/r disease. A total of 33 patients (57%) received CD19-directed CAR-T, with a median interval of 4 months (range: 1–31) between CAR-T infusion and subsequent initiation of BsAbs. Overall, 33 patients (57%) were refractory to the last treatment prior to initiation of BsAb therapy. The median LDH value at the start of treatment was 270 U/l (range: 131–1097 U/l), and 10% of patients had a bulky disease (> 7.5 cm). A total of 45 patients (78%) and 13 patients (22%) received glofitamab and epcoritamab, respectively.

**Table 1:** Characteristics of patients analyzed in the study

Parameters	Patients
Number of patients	58
Age at lymphoma diagnosis, median in y (range)	62 (27–81)
IPI at diagnosis, median (range)	2 (1–4)
Gender, n (%)	
Male	39 (67%)
Female	19 (33%)
Lymphoma types, n (%)	
DLBCL	42 (72%)
- GCB	19 (45%)
- Non-GCB	9 (21%)
- Unclassified	14 (33%)
HGBL	16 (28%)
- NOS	4 (25%)
- BCL2/6, Myc rearr.	11 (69%)
- 11q del.	1 (6%)
Bulky dz (> 7,5 cm) prior to BsAb, n (%)	6 (10%)
LDH [U/l] prior to BsAb, median (range)	270 (131–1097)
Therapies prior BsAb	
Median therapy lines prior BsAb, n (range)	3 (1–8)
Anti-CD20/anthracycline-based reg., n (%)	58 (100%)
Platinum-based salvage reg., n (%)	44 (76%)
Prior bendamustin application, n (%)	19 (33%)
Prior autologous SCT, n (%)	10 (17%)
Prior CAR-T, n (%)	33 (57%)
CAR-T product, n (%)	
Axi-cel	24 (73%)
Liso-cel	3 (9%)
Tisa-cel	6 (18%)
Median time (mo.) to relapse after CAR-T infusion, n (range)	3 (1–31)
Median time (mo.) between CAR-T and BsAb, n (range)	4 (1–31)
Refractory to last tx prior BsAb, n (%)	33 (57%)
IPI at initiation of BsAb, n (%) available for 36/58 cases	
Low (0–1)	9 (25%)
Intermediate (2–3)	19 (53%)
High (4–5)	8 (22%)
ECOG at initiation of BsAb, n (%) available for 51/58 cases	
ECOG 0–2	49 (96%)
ECOG > 2	2 (4%)
BsAb type	
Glofitamab, n (%)	45 (78%)
- cycles, median (range)	4 (1–12)
Epcoritamab, n (%)	13 (22%)
- cycles, median (range)	3 (1–10)

*y* year, *IPI* International Prognostic Index, *DLBCL* diffuse large B-cell lymphoma, *GCB* germinal center B-cell-like, *Non-GCB* non germinal center B-cell-like, *HGBL* high grade B-cell lymphoma, *NOS* not otherwise specified, *rearr* rearrangement, *del* deletion, *dz* disease, *BsAb* bispecific antibodies, *LDH* lactate dehydrogenase, *reg* regime, *SCT* stem cell transplantation, *CAR-T* chimeric antigen receptor T-cell therapy, *Axi-cel* axicabtagen-Ciloleucel, *Liso-cel* lisocabtagen maraleucel, *Tisa-cel* tisagenlecleucel, *mo* months, *ECOG* eastern cooperative oncology group performance status

### Outcomes of treatment with BsAb

The outcomes of BsAb therapy are summarized in Table 2. The median follow-up after initiation of BsAb was seven months (range: 0–52 months). 44% of patients (26/58) responded to BsAb, 22% of patients achieved a CR (13/58) while 21% of patients responded with a PR (12/58).

**Table 2:** Outcomes of BsAb therapy

Parameters	Patients	
Best overall response rate under BsAb (CR, PR), n (%)	26 (44%)	
Reason for ending BsAb, n (%)		
PD	32 (55%)	
End of treatment (EOT)	10 (17%)	
Others	4 (7%)	
Ongoing	2 (3%)	
Toxicity	3 (5%)	
Subsequent therapy after BsAb, n (%)	33 (57%)	
Median follow-up from BsAb initiation, mo. (range)	7 (0–52)	
Relapsed/refractory disease post-BsAb, n (%)	33 (57%)	
Remisson status last follow up, n (%)		
CR		13 (22%)
PR		12 (21%)
SD		1 (2%)
PD		32 (55%)
Survival status at last follow-up, n (%)		
alive	29 (50%)	
dead	29 (50%)	
Cause of death, n (%)		
r/r lymphoma	20 (34%)	
Non-relapse mortality	5 (9%)	
- Infection	4 (80%)	
- Second malignancy	1 (20%)	
unknown	4 (7%)	

*CR* complete remission, *PR* partial remission, *SD* stable disease, *PD* progressive disease, *BsAb* bispecific antibodies, *mo* month, *r/r lymphoma* relapse/refractory lymphoma

Reasons for ending therapy with BsAb were disease progression (32/58; 55%), planned end of treatment with glofitamab (12 cycles; 10/58; 17%) and other reasons (4/58; 7%). Overall, 57% of the patients (33/58) received subsequent therapies (Table 2).

**Fig. 2:**
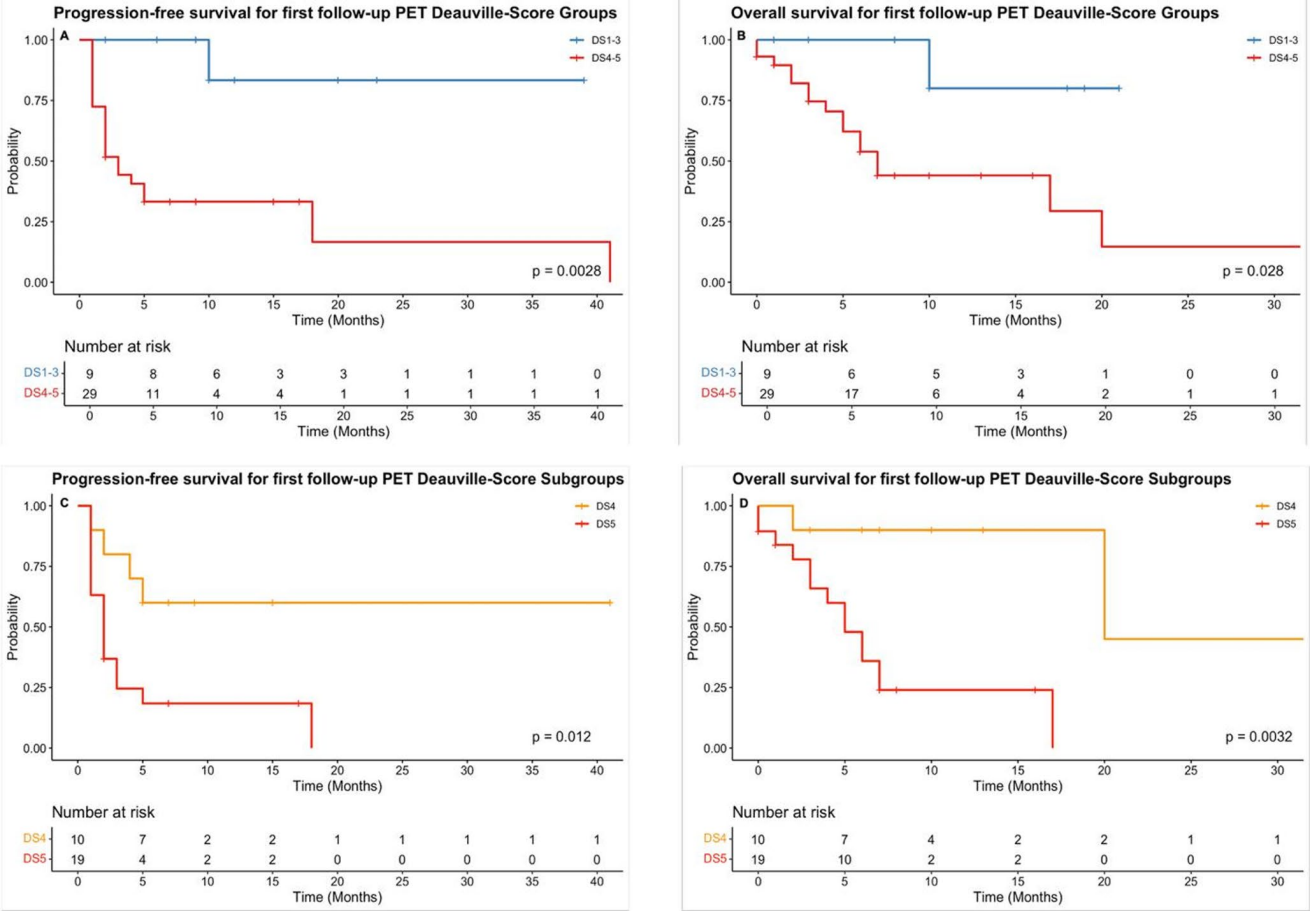
Kaplan-Meier analysis of Deauville-Score groups in the first PET after BsAb initiation. PET/CT were performed after a median of 1.9 months (IQR). DS 1–3 or DS 4–5 were correlated with (**A**) PFS or (**B**) OS and DS4-5 further subdivided into DS4 and DS5 for more granular (**C**) PFS and (**D**) OS of this high-risk group

At the last follow-up, 50% were alive. Reasons for death were relapse/progression (34%; 20/58) and non-relapse mortality (9%; 5/58) due to infection (4/5) and second malignancy (1/5). Median PFS and median OS were 3 months (range: 0.4–53 months) and 7 months (range: 0.5–53 months), respectively (Fig. 2S). Notably, patients with CMR demonstrated excellent survival with 1-year PFS of 83% and OS of 80% following initiation of BsAb treatment after a median follow-up of 20 months.

### PET scanner variability

**Fig. 3:**
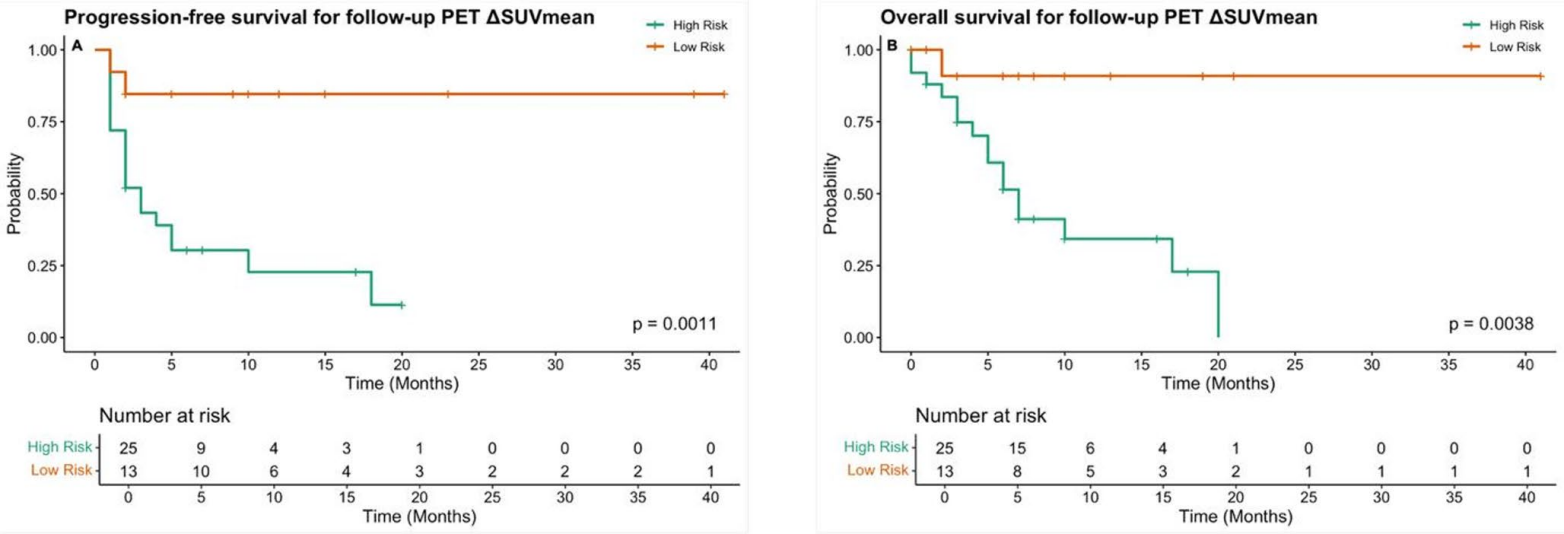
Kaplan-Meier analysis of MSLRS binarized ΔSUVmean in the first PET after BsAb initiation. PET/CT were done after a median of 1.9 months post-BsAb initiation and patients separated by the identified cut-off values for ΔSUVmean for high-risk towards shorter PFS (**A**) or OS (**B**) respectively

Across the analyses, SUVmax, SUVpeak, SUVmean, whole-body MTV, and whole-body TLG showed no significant scanner dependency (all global *P* > 0.05 with small effect sizes: ε² range = −0.010–0.091). In contrast, SUVmin demonstrated consistent scanner-related differences at baseline (ε² = 0.364, *P* < 0.001) and at first follow-up after BsAb treatment initiation (ε² = 0.231, *P* = 0.004) primarily driven by lower values in the Essen cohort (Tables 1S, 2S and 3S, Fig. 3S).

#### Baseline FDG-PET

In the ideal cut-off analysis (MSLRS) for PFS and OS events all baseline PET parameters achieved significant prognostic value after binarized risk-group stratification in Kaplan-Meier analysis (all *P* < 0.05). In Cox proportional regression analysis for OS events the determined low-risk group of SUVmax and SUVmean revealed no events and calculation of hazards was not feasible. Therefore, SUVpeak (instead of SUVmax) and whole-body MTV was selected and described in greater detail to remain closely aligned with clinical conditions. Patients with SUVpeak values > 7.9 (high-risk, *n* = 47/58, 81%) exhibited a markedly increased hazard of progression (HR = 6.15, *P* = 0.0127; C-index = 0.89) and death (HR = 4.72, *P* = 0.0406; C-index = 0.95) with a median PFS of 2 months vs. not reached (*P* = 0.0036, Fig. 1A) and median OS of 8 vs. 21 months (*P* = 0.026, Fig. 1B). The volumetric parameter whole-body MTV delineated a smaller group of high-risk patients (*n* = 7/58, 12%) with whole-body MTV > 504.7 cm³, who experienced a significantly elevated risk of progression (HR = 5.6, *P* = 0.00025; C-index = 0.94). Of these 7 patients, 6 had diffuse bone marrow involvement and 1 patient revealed diffuse peritoneal involvement, which led to the high whole-body MTV. This group showed a median PFS of 1 vs. 3 months (*P* = 0.00016; Fig. 1C). Notably, all patients in the whole-body MTV high-risk group progressed within 2 months, whereas 41% of those in the low-risk group remained progression-free beyond 15 months. Patients with bulky disease reached a median PFS of 1 month, while those without demonstrated a median PFS of 4.5 months (HR = 6.97, *P* < 0.001). In the same lines, the median OS was 3.3 vs. 11.6 months (HR = 4.88, *P* = 0.002) in both groups, accordingly. In the ideal cut-off analysis (MSLRS) for OS events, patients with whole-body MTV values > 39.1 cm^3^ (high-risk, *n* = 35/58, 60%) exhibited a markedly increased hazard of death (HR = 2.52, *P* = 0.0259) with a significantly reduced median OS of 7 vs. 21 months (*P* = 0.021, Fig. 1D). Time-independent ROC–AUC analysis performed for verification identified different cut-off values for the aforementioned PET parameters. While these cut-offs provided only limited survival discrimination in Kaplan–Meier analysis, SUVpeak, SUVmin, and whole-body TLG demonstrated statistically significant associations with survival (all *P* < 0.05, Table 4S). Time-fixed ROC–AUC analysis of baseline PET parameters (cut-offs: PFS 6 months, OS 12 months) also demonstrated significant risk stratification. However, the discriminative performance was lower than that observed with the MSLRS-based analysis (Tables 5S, 6S and 7S). The detailed ideal cut-off analysis (MSLRS) of baseline PET parameters is demonstrated in Table 3; Fig. 3S.

**Table 3:** Detailed statistical analysis of baseline PET; indicated parameters were binarized using MSLRS to groups with maximal p and shown is the best-fit cut-off value with patients at higher risk regarding high-risk for survival events, progression-free and for overall survival

Progression-free survival							
Parameter	*n* = 58	Cut-off	Log-rank *P*	HR	95%CI	*P*	C-index
SUVpeak	47 (81%)	> 7.9	0.0036	6.15	1.47–25.65	**0.0127**	0.89
SUVmax	50 (86%)	> 9.62	0.011	8.02	1.1–58.74	**0.0404**	0.9
SUVmean	50 (86%)	> 5.52	0.011	8.02	1.1–58.74	**0.0404**	0.9
SUVmin	42 (72%)	> 4	0.00063	5.06	1.78–14.37	**0.0024**	0.88
whole-body MTV	7 (12%)	> 504.7	0.00016	5.66	2.24–14.29	**0.00025**	0.94
whole-body TLG	14 (24%)	> 3599.9	0.00026	3.84	1.86–7.91	**0.00027**	0.87
Overall survival							
Parameter	*n* = 58	Cut-off	Log-rank P	HR	95%CI	P	C-index
SUVpeak	47 (81%)	> 7.9	0.026	4.72	1.07–20.87	**0.0406**	0.95
SUVmax	50 (86%)	> 9.62	0.02	N/A	N/A	N/A	N/A
SUVmean	50 (86%)	> 5.52	0.02	N/A	N/A	N/A	N/A
SUVmin	39 (67%)	> 4.04	0.0028	5.1	1.54–16.89	**0.0077**	0.84
whole-body MTV	35 (60%)	> 39.1	0.021	2.52	1.12–5.68	**0.0259**	0.76
whole-body TLG	20 (34%)	> 1508.5	0.0043	2.98	1.36–6.51	**0.00062**	0.79

*HR* hazard ratio, *CI* confidence interval, *C-Index* Concordance index, *SUV* standardized uptake values, *N/A* not available, *whole-body MTV* metabolic tumor volume, *whole-body TLG* total lesion glycolysis

#### First follow-up FDG-PET after BsAb initation

A total of 45/58 patients (78%) met the criteria for the first PET after BsAb initiation analysis while 7/45 patients (16%) were excluded due to progressive disease. Median time from the initiation of BsAb to the first PET after BsAb initiation was 1.9 months (range: 0.7–8.2 months).

In the PFS analysis, patients in the DS4–5 group (*n* = 29/38, 76%) compared to the DS1–3 group (*n* = 9/38, 24%), exhibited a significantly higher hazard of disease progression (HR = 11.42, *P* = 0.0182, C-index = 0.97), with a median PFS of 3 months (*P* = 0.0028; Fig. 2A), whereas the median PFS was not reached in the DS1–3 group. For OS, Kaplan–Meier analysis revealed an inferior outcome for DS4–5 patients, with a median OS of 7 months (*P* = 0.028), while the DS1–3 group did not reach median OS within the observation period (Fig. 2B). Noteworthy, subgroup analysis of patients with DS5 (*n* = 19/29, 66%) compared to DS4 (*n* = 10/29, 35%) demonstrated a significantly higher hazard of progression (HR = 3.8, *P* = 0.019) and death (HR = 11.4, *P* = 0.001) for DS5 group. Particularly, median PFS was 2 months vs. not reached (*P* = 0.012; Fig. 2C), while median OS was 5 vs. 20 months (*P* = 0.0032; Fig. 2D). The 1-year survival rate for DS1-3 vs. DS4-5 was significantly lower in the DS4-5 group with 83% vs. 33% for PFS, and 80% vs. 44% for OS (both *P* < 0.01), respectively.

Of the DS4 group (10/29, 34%), 3 out of 10 converted into CR in the most recent follow-up. The remaining patients presented with either ongoing PR (5/10) or PD (2/10) at the last follow-up. In the group with DS5 (19/29, 66%), 5% achieved CR later (1/19), whereas 85% presented with PD (16/19) and 2 out of 19 with PR at the last follow-up.

To allow inclusion of DS1–3 patients (*n* = 9/38, 24%) in the risk stratification analysis, a VOI using a 41%-SUVmax threshold was placed within the area of highest FDG uptake on the baseline PET scan. The resulting median values (range) were as follows: SUVpeak 1.92 (0.64–2.98), SUVmax 2.05 (0.79–3.23), SUVmean 1.67 (0.54–2.63), SUVmin 0.93 (0.24–1.23), whole-body MTV 2.07 cm³ (0.5–4.07), and whole-body TLG 3.6 (0.62–8.49).

In the ideal cut-off analysis (MSLRS) of ΔPET parameters, ΔSUVmean emerged as the most powerful predictor of outcome in comparison of C-index values. Patients who failed to achieve a reduction of at least 75.5% in binarized ΔSUVmean (*n* = 25/38, 66%) had a markedly increased risk of progression (HR = 7.65, *P* = 0.0065; C-index = 0.92) and death (HR = 11.1, *P* = 0.001; C-index = 0.95), with a median PFS of 3 months (*P* = 0.0011 Fig. 3A) and a median OS of 7 months respectively (*P* = 0.0038; Fig. 3B). In contrast, both median PFS and median OS were not reached during the observation period for patients at lower risk (*n* = 13/38, 34%). The 1-year survival rate for low-risk and high-risk ΔSUVmean groups was significantly better for the former with 85% vs. 23% for PFS, and 91% vs. 34% for OS (both *P* < 0.01), respectively. Time-independent ROC–AUC analysis performed for verification yielded slightly different cut-off values for the aforementioned PET parameters. Although survival discrimination was inferior to that obtained by MSLRS analysis, all PET parameters demonstrated statistically significant associations with survival (all *P* < 0.05, Table 8S). Time-fixed ROC–AUC analysis for PET parameters of the first PET after BsAb initiation (cut-offs: PFS 6 months, OS 12 months) also demonstrated significant risk stratification. However, the discriminative performance was lower than that observed with the MSLRS-based analysis (Tables 9S, 10S and 11S).

Comparison of Cox regression models for DS and ideal cut-off (MSLRS) analysis demonstrated comparable discriminatory power for PFS, with no significant difference between the two approaches (C-index: DS = 0.97 vs. MSLRS = 0.92; *P* = 0.21). In contrast, the ideal cut-off (MSLRS) analysis provided a significantly superior discriminatory ability for OS events (C-index: DS = 0.72 vs. MSLRS = 0.95; *P* = 0.02). The detailed ideal cut-off analysis (MSLRS) is demonstrated in Table 4; Fig. 4S.

**Table 4:** Detailed statistical analysis of first first PET after BsAb initiation showing MSLRS-binarized cut-offs to group patients for high-risk for shorter progression-free or overall survival including multivariate comparison using Cox regression

Progression-free survival							
Parameter	*n* = 38	Cut-off	Log-rank *P*	HR	95%CI	*P*	C-index
DS4-5	29 (76%)	N/A	0.0028	11.42	1.51–86.28	**0.0182**	0.97
ΔSUVpeak	26 (68%)	>−0.782	0.0024	6.89	1.6–29.72	**0.0096**	0.84
ΔSUVmax	24 (63%)	>−0.785	0.0021	5.47	1.6–18.7	**0.0068**	0.85
ΔSUVmean	25 (66%)	>−0.755	0.0011	7.65	1.77–33.08	**0.0065**	0.92
ΔSUVmin	23 (61%)	>−0.591	0.0015	5.62	1.64–19.2	**0.0059**	0.81
Δwhole-body MTV	6 (16%)	> 1.64	0.0029	4.26	1.53–11.85	**0.0054**	0.81
Δwhole-body TLG	21 (55%)	>−0.861	0.0098	3.41	1.24–9.33	**0.0171**	0.76
Overall survival							
Parameter	**n**	**Cut-off**	**Log-rank P**	**HR**	**95%CI**	**P**	**C-index**
DS4-5	29 (76%)	N/A	0.028	7.07	0.93–53.85	0.059	0.72
ΔSUVpeak	18 (47%)	>−0.378	0.0068	4.19	1.36–12.93	**0.0127**	0.8
ΔSUVmax	15 (40%)	>−0.141	0.0026	4.38	1.54–12.5	**0.00574**	0.81
ΔSUVmean	25 (66%)	>−0.755	0.0038	11.1	1.45–84.69	**0.0013**	0.95
ΔSUVmin	22 (58%)	>−0.545	0.0031	6.89	1.56–30.36	**0.0107**	0.85
Δwhole-body MTV	13 (34%)	> 0.126	0.016	3.35	1.18–9.48	**0.0228**	0.85
Δwhole-body TLG	19 (50%)	>−0.779	0.0055	4.93	1.41–17.25	**0.0124**	0.79

*HR* hazard ratio, *C-Index * Concordance index, *SUV* standardized uptake values, *DS* Deauville-score, *N/A* not available, *whole-body MTV* metabolic tumor volume, *whole-body TLG* total lesion glycolysis

An additional sensitivity analysis was performed, applying PFS-derived cutoffs to OS, on the basis that PET-derived parameters primarily reflect tumor biology and treatment response and are more closely aligned with PFS than OS.

This unified approach revealed that PET-derived parameters maintained a strong association with overall survival (OS), while PFS-derived cutoffs exhibited a diminished discriminatory capacity in comparison to OS-optimized cutoffs. Prior to the initiation of BsAb therapy, the cut-off values for PFS and OS exhibited near-identical thresholds for SUVpeak, SUVmax, and SUVmean, while disparities were apparent for MTV and TLG. Separate analyses were conducted for these parameters in baseline PET, as well as for all parameters from the first PET after BsAb initiation (Tables 14S, 15S, 16S and 17S).

### Univariate and multivariate analysis with exploratory Cox regression model

In univariate Cox regression analysis for PFS, bulky disease (HR = 3.03, *P* = 0.026), LDH level (interquartile range [IQR]-adjusted HR = 1.46, *P* = 0.005), ECOG ≥ 2 (ECOG 2: HR = 11.51, *P* < 0.001; ECOG 3: HR = 6.16, *P* = 0.029), and baseline whole-body MTV (IQR-adjusted HR = 1.34, *P* = 0.001) were associated with shorter PFS. In multivariate analysis, baseline whole-body MTV remained the only independent predictor of PFS (HR = 1.55, *P* = 0.028). Detailed analysis is demonstrated in Table 12S.

For OS, univariate analysis identified bulky disease (HR = 4.15, *P* = 0.005), extranodal disease (HR = 2.43, *P* = 0.030), LDH level (IQR-adjusted HR = 1.66, *P* < 0.001), ECOG 2 (HR = 6.54, *P* = 0.010), ECOG 3 (HR = 12.88, *P* = 0.005), and baseline whole-body MTV (IQR-adjusted HR = 1.34, *P* = 0.003) as significant predictors. In multivariate analysis, ECOG 2 (HR = 6.89, *P* = 0.027) and baseline whole-body MTV (HR = 1.82, *P* = 0.010) remained independently associated with OS. Detailed analysis is demonstrated in Table 13S.

Using the predefined risk model (bulky disease, extranodal involvement, LDH level, ECOG and whole-body MTV; *n* = 47/58) dichotomization at the optimal cut-off significantly stratified outcomes. For PFS, the cut-off of 0.6319 separated patients into distinct risk groups (log-rank *p* < 0.001), with high-risk patients showing inferior PFS (HR 2.38, 95% CI 1.11–5.10; *p* = 0.026, Fig. 6S). Similarly, for OS, the cut-off of 0.4126 yielded significant risk discrimination (log-rank *p* < 0.001). High-risk patients demonstrated markedly worse OS (HR 5.83, 95% CI 2.22–15.27; *p* < 0.001, Fig. 7S).

## Discussion

This multicenter retrospective study investigates the prognostic role of longitudinal FDG-PET parameters at baseline and first follow-up in patients with r/r LBCL treated with BsAb. Optimal cut-off analysis of binarized PET-derived metrics at BsAb initiation effectively identified patients with high metabolic activity and/or tumor burden who were at increased risk of subsequent progression and death. In fact, patients with CMR demonstrated excellent survival with a median PFS and OS that was not reached after a median observation of 20 months. Our findings highlight the potential of FDG-PET to enable risk-adapted treatment stratification in a population with limited therapeutic options.

The variability of the scanner was confined to SUVmin, a parameter that has been shown to be highly sensitive to image noise, reconstruction settings and VOI boundary effects. The stability of all other PET metrics (SUVmax, SUVpeak, SUVmean, whole-body MTV, whole-body TLG) has been confirmed, thereby ensuring cross-center comparability.

At baseline, elevated SUV-based and volumetric PET metrics identified high-risk subgroups. Specifically, the high-risk group of binarized SUVpeak and whole-body MTV values were associated with markedly shorter PFS. All patients classified as high risk by whole-body MTV progressed within two months, whereas 41% of low-risk patients remained progression-free beyond 15 months. These observations underscore tumor burden and metabolic activity as markers of resistance to BsAbs. Furthermore, SUV-based metrics (SUVpeak) and volumetric markers (whole-body MTV) provided complementary prognostic information for OS outcomes. Integration with clinical risk factors (bulky disease, extranodal involvement, LDH level, ECOG) improved the stratification of PFS and OS, thereby highlighting the value of combined metabolic and clinical risk models.

Taken together, these results are consistent with prior reports in r/r LBCL including CAR-T cohorts demonstrating that standard and volumetric PET parameters are robust predictors of survival for BsAb treatment as well [23–27].

At first FDG-PET after BsAb initiation, PET metrics effectively stratified risk among patients without confirmed progression. Patients with CMR demonstrated excellent survival with 1-year PFS of 83% and OS of 80% following initiation of BsAb treatment and after a median follow-up of 20 months. Notably, individuals with non-CMR (DS4–5) but still no progression at first follow-up, had significantly shorter PFS and OS with 1-year PFS and OS of 33% and 44%, only. Subgroup analysis in DS-4–5 cohort showed particularly poor outcomes for DS5, whereas DS4 exhibited more favorable survival, indicating a clinically meaningful distinction between DS4 and DS5. These findings highlight the value of early PET response assessment for prognostication and treatment guidance. Beyond visual assessment at first FDG-PET after BsAb initiation, semi-quantitative PET dynamics, especially binarized ΔSUVmean groups, provided robust discrimination for both PFS and OS. Failure to achieve a ≥ 75% reduction in ΔSUVmean was associated with a markedly increased hazard of death (HR = 11.1), underscoring the importance of early metabolic change as a surrogate of BsAb treatment efficacy. Notably, ΔSUVmean outperformed DS groups for OS prediction and offered comparable stratification for PFS, suggesting that semi-quantitative PET-derived markers may augment, or even surpass, visual response criteria in this setting.

Our results are in line with other studies reporting that non-CMR patients after initiation of BsAb have a poor prognosis, while CMR patients show promising survival rates [6–9]. In the non-CMR cohort, a small subset of patients demonstrated a transition from partial remission in the first FDG-PET after BsAb initiation to complete remission during further follow-up, suggesting that therapeutic responses can continue to deepen over time. However, the majority of non-CMR patients at first PET after BsAb initiation developed a r/r disease later which was reflected in better granularity when split for DS4 and DS5, with all of D5 patients relapsing.

In the view of the markedly inferior prognoses indicated by DS4-5 in the first FDG-PET after BsAb initiation, it is evident that adjustments or additions to the treatment may be required to improve prognosis. While non-CMR patients on BsAb and still CAR-T naive patients can be consolidated with subsequent CAR-T, CAR-T-pretreated patients potentially can be treated with an allogenic transplantation.

This multicenter study has limitations inherent to retrospective designs and due to the sample size. Therefore, stratified analyses by BsAbs (glofitamab vs. epcoritamab) were not performed. The timing of the first FDG-PET after BsAb initiation varied, with a median of 1.9 months, corresponding to approximately two cycles of both epcoritamab and glofitamab. However, the range of 0.7–8.2 months reflects variability in real-world practice, and the specific clinical reasons for earlier or delayed imaging were not systematically collected.

The selection of lymphoma-related FDG uptake on PET scans was performed and carefully evaluated by the PET readers. However, physiological uptake, treatment-related activation (e.g., bone marrow activation), or other non–lymphoma-related uptake may also have been segmented, potentially leading to an overestimation of the whole-body MTV.

Recent international consensus recommendations suggest a fixed SUV cutoff of 4 for lymphoma segmentation. Nevertheless, this recommendation was principally developed for baseline PET imaging, where FDG uptake is ordinarily more homogeneous. Conversely, subsequent scans frequently exhibit heterogeneous uptake patterns, particularly in heavily pretreated patients. In order to account for this, a relative SUVmax-based threshold of 41% was applied, thus allowing more flexible delineation of metabolically active tumor regions under heterogeneous conditions. In CMR patients (DS1–3), segmentation was based on the region of maximum baseline PET uptake, as a fixed SUV cutoff of 4 would not permit meaningful segmentation in this context.

Treatment heterogeneity, including extensive prior therapies and variability in management and imaging protocols, may confound associations and complicate interpretation. Nevertheless, the consistently high C-indices values across most FDG-PET parameters support the stability of our findings. Prospective validation in larger, harmonized cohorts, ideally within clinical trials that incorporate standardized FDG-PET acquisition/analysis and FDG-PET-guided decision-making, is warranted.

## Conclusion

The present study demonstrates that both baseline and early FDG-PET metrics are strong predictors of treatment outcomes in r/r LBCL patients receiving BsAbs. The integration of FDG-PET-derived risk stratification into clinical practice has the potential to facilitate tailoring of therapy intensity, optimization of sequencing strategies, considerations for early relapse/refractoriness, and in the long run, to improve survival of high-risk patients.

## Supplementary Information

Below is the link to the electronic supplementary material.

Supplementary File 1 (DOCX 1.52 MB)

## Data Availability

The datasets generated during and/or analysed during the current study are available from the corresponding author on reasonable request.
