# Supplementary material for: Prognostic role of FDG-PET in patients with relapsed/refractory large B-cell lymphoma treated with CD3-CD20 directed bispecific antibodies: a multicentric analysis
Source: Eur J Nucl Med Mol Imaging. 2026 Jun 1;53(10):5982–93. doi: 10.1007/s00259-026-07958-4 (PMC13421258; doi:10.1007/s00259-026-07958-4)
Supplement: Supplementary file 1 — (DOCX 1.52 MB) [file 259_2026_7958_MOESM1_ESM.docx]

**SUPPLEMENTS**

**Table 1S:** Scanner-related variability of PET parameters

| **Time point** | **Parameter** | **n** | **Groups** | **Kruskal–Wallis χ²** | **df** | **p-value** | **ε²** | **Linear model p_global** |
| --- | --- | --- | --- | --- | --- | --- | --- | --- |
| **Baseline** | SUVmax | 58 | 4 | 2.51 | 3 | 0.473 | −0.009 | 0.391 |
|  | SUVpeak | 58 | 4 | 2.44 | 3 | 0.486 | −0.010 | 0.223 |
|  | SUVmean | 58 | 4 | 5.17 | 3 | 0.160 | 0.040 | 0.354 |
|  | **SUVmin** | 58 | 4 | **22.7** | 3 | **<0.001** | **0.364** | **<0.001** |
|  | MTV | 58 | 4 | 5.12 | 3 | 0.163 | 0.039 | 0.162 |
|  | TLG | 58 | 4 | 4.26 | 3 | 0.234 | 0.023 | 0.173 |
| **Follow-up** | SUVmax | 38 | 3 | 3.10 | 2 | 0.212 | 0.032 | 0.184 |
|  | SUVpeak | 38 | 3 | 2.99 | 2 | 0.224 | 0.028 | 0.155 |
|  | SUVmean | 38 | 3 | 5.17 | 2 | 0.075 | 0.091 | 0.080 |
|  | **SUVmin** | 38 | 3 | **10.1** | 2 | **0.006** | **0.231** | **0.004** |
|  | MTV | 38 | 3 | 3.73 | 2 | 0.155 | 0.050 | 0.177 |
|  | TLG | 38 | 3 | 4.16 | 2 | 0.125 | 0.062 | 0.106 |

**Table 2S:** Scanner coefficients from linear regression (reference: Münster)

| **Parameter** | **Scanner** | **β (log-scale)** | **Standard error** | **p-value** |
| --- | --- | --- | --- | --- |
| **Baseline SUVmax** | Bern | −0.119 | 0.195 | 0.542 |
|  | Essen | 0.095 | 0.186 | 0.611 |
|  | Erlangen | 0.426 | 0.308 | 0.172 |
| **Baseline SUVpeak** | Bern | −0.201 | 0.218 | 0.360 |
|  | Essen | 0.097 | 0.208 | 0.644 |
|  | Erlangen | 0.550 | 0.345 | 0.116 |
| **Baseline SUVmean** | Bern | −0.126 | 0.169 | 0.458 |
|  | Essen | −0.261 | 0.161 | 0.111 |
|  | Erlangen | −0.313 | 0.267 | 0.247 |
| **Baseline SUVmin** | Bern | −0.088 | 0.203 | 0.667 |
|  | **Essen** | **−1.08** | 0.194 | **<0.001** |
|  | Erlangen | 0.130 | 0.321 | 0.686 |
| **Baseline MTV** | Bern | −1.14 | 0.592 | 0.060 |
|  | Essen | 0.203 | 0.565 | 0.721 |
|  | Erlangen | −0.814 | 0.936 | 0.389 |
| **Baseline TLG** | Bern | −1.27 | 0.660 | 0.060 |
|  | Essen | −0.059 | 0.630 | 0.926 |
|  | Erlangen | −1.38 | 1.04 | 0.191 |

**Table 3S:** Scanner coefficients from linear regression (reference: Münster)

| **Parameter** | **Scanner** | **β (log-scale)** | **Standard error** | **p-value** |
| --- | --- | --- | --- | --- |
| **Follow-up SUVmax** | Bern | −0.600 | 0.486 | 0.225 |
|  | Essen | −0.753 | 0.426 | 0.086 |
| **Follow-up SUVpeak** | Bern | −0.640 | 0.456 | 0.169 |
|  | Essen | −0.723 | 0.400 | 0.080 |
| **Follow-up SUVmean** | Bern | −0.620 | 0.449 | 0.176 |
|  | Essen | −0.882 | 0.393 | 0.032 |
| **Follow-up SUVmin** | Bern | −0.599 | 0.447 | 0.189 |
|  | **Essen** | **−1.40** | 0.392 | **0.001** |
| **Follow-up total bodyMTV** | Bern | −0.947 | 0.906 | 0.303 |
|  | Essen | −1.48 | 0.795 | 0.071 |
| **Follow-up total-body TLG** | Bern | −1.57 | 1.27 | 0.224 |
|  | Essen | −2.36 | 1.11 | 0.041 |

**Table 4S:** Comparison of ROC and MSLRS cut-off analysis for baseline PET parameters

| **PET-Parameter / PFS** | **AUC** | **AUC P** | **ROC**  **Cut-Off** | **KM P** | **HR** | **Cox P** | **MSLRS Cut-Off** | **KM P** | **HR** | **Cox P** |
| --- | --- | --- | --- | --- | --- | --- | --- | --- | --- | --- |
| SUVmax | 0.68 | 0.02 | >17.63 | 0.51 | 2.13 | 0.091 | >9.62 | 0.011 | 8.02 | 0.0404 |
| SUVpeak | 0.63 | 0.12 | >8.37 | 0.004 | 5.38 | 0.021 | >7.6 | 0.0036 | 6.15 | 0.0127 |
| SUVmean | 0.69 | 0.016 | >10.32 | 0.049 | 1.98 | 0.088 | >5.52 | 0.011 | 8.02 | 0.0404 |
| SUVmin | 0.76 | 0.001 | >7.05 | 0.006 | 2.38 | 0.018 | >4 | 0.00063 | 5.06 | 0.0024 |
| whole-body MTV | 0.64 | 0.057 | >39.66 | 0.085 | 1.72 | 0.13 | >504.7 | 0.00016 | 5.66 | 0.00025 |
| whole-body TLG | 0.68 | 0.016 | >1553.56 | 0.001 | 2.53 | 0.007 | >3599.9 | 0.00026 | 3.84 | 0.00027 |
| **PET-Parameter / OS** | **AUC** | **AUC**  **P** | **ROC**  **Cut-Off** | **KM P** | **HR** | **Cox P** | **MSLRS Cut-Off** | **KM P** | **HR** | **Cox P** |
| SUVmax | 0.63 | 0.09 | >10.42 | 0.02 | N/A* | N/A* | >7.9 | 0.02 | N/A* | N/A* |
| SUVpeak | 0.56 | 0.46 | >8.37 | 0.543 | 1.34 | 0.55 | >9.62 | 0.026 | 4.72 | 0.0406 |
| SUVmean | 0.64 | 0.049 | >6.09 | 0.02 | N/A* | N/A* | >5.52 | 0.02 | N/A* | N/A* |
| SUVmin | 0.72 | 0.001 | >4.01 | 0.008 | 5.49 | 0.02 | >4.04 | 0.0028 | 5.1 | 0.0077 |
| whole-body MTV | 0.57 | 0.33 | >39.66 | 0.021 | 2.48 | 0.03 | >39.1 | 0.021 | 2.52 | 0.0259 |
| whole-body TLG | 0.6 | 0.21 | >42.61 | 0.026 | N/A* | N/A* | >1508.5 | 0.0043 | 2.98 | 0.00062 |

*No events in the low-risk group

PET = Positron-Emission-Tomography; PFS = Progression-free survival; AUC = Area under the curve; ROC = Receiver operating characteristics; KM = Kaplan-Meier; HR = Hazard-Ratio; Cox (Cox proportional regression analysis); MSLRS = maximally selected long-rank statistical analysis;

**Table 5S:** ROC-AUC analysis of baseline PET parameters for prediction of progression-free survival after 6 months

| **Parameter** | **N** | **Events** | **AUC** | **95% CI** | **p-value** | **Cut-off** | **Sensitivity** | **Specificity** | **Youden J** |
| --- | --- | --- | --- | --- | --- | --- | --- | --- | --- |
| SUVmin | 58 | 40 | 0.665 | 0.511–0.818 | 0.339 | 4.06 | 0.775 | 0.556 | 0.331 |
| TLG | 58 | 40 | 0.635 | 0.483–0.786 | 0.491 | 3021 | 0.350 | 0.944 | 0.294 |
| MTV | 58 | 40 | 0.621 | 0.467–0.774 | 0.575 | 39.7 | 0.675 | 0.556 | 0.231 |
| SUVmean | 58 | 40 | 0.590 | 0.425–0.756 | 0.745 | 9.00 | 0.750 | 0.500 | 0.250 |
| SUVmax | 58 | 40 | 0.568 | 0.404–0.732 | 0.895 | 16.9 | 0.800 | 0.389 | 0.189 |
| SUVpeak | 58 | 40 | 0.544 | 0.368–0.721 | 0.947 | 8.37 | 0.875 | 0.333 | 0.208 |

PET = Positron-Emission-Tomography; PFS = Progression-free survival; AUC = Area under the curve; ROC = Receiver operating characteristics;

**Table 6S:** ROC-AUC analysis of baseline PET parameters for prediction of overall survival at 12 months

| **Parameter** | **N** | **Events** | **AUC** | **95% CI** | **p-value** | **Cut-off** | **Sensitivity** | **Specificity** | **Youden J** |
| --- | --- | --- | --- | --- | --- | --- | --- | --- | --- |
| MTV | 58 | 46 | 0.725 | 0.566–0.884 | 0.104 | 57.2 | 0.587 | 0.917 | 0.504 |
| TLG | 58 | 46 | 0.688 | 0.519–0.857 | 0.206 | 1005 | 0.543 | 0.917 | 0.460 |
| SUVmin | 58 | 46 | 0.595 | 0.420–0.770 | 0.719 | 7.50 | 0.565 | 0.750 | 0.315 |
| SUVmax | 58 | 46 | 0.578 | 0.417–0.738 | 0.775 | 27.3 | 0.435 | 0.917 | 0.351 |
| SUVpeak | 58 | 46 | 0.547 | 0.360–0.734 | 0.946 | 13.8 | 0.674 | 0.500 | 0.174 |
| SUVmean | 58 | 46 | 0.489 | 0.300–0.679 | 0.726 | 11.1 | 0.609 | 0.500 | 0.109 |

PET = Positron-Emission-Tomography; PFS = Progression-free survival; OS = Overall survival; ROC = Receiver operating characteristics;

**Table 7S:** Kaplan-Meier survival analysis, Cox-Regression and C-Index evaluation for baseline PET predicting progression-free survival and overall survival after 6 and 12 months

| **Baseline PET – Progression-free survival 6 months – ROC Cut-Off** | | | | | | | |
| --- | --- | --- | --- | --- | --- | --- | --- |
| **Parameter** | **n = 58**  **High-Risk** | **Cut-off** | **Log-rank P** | **HR** | **95%CI** | **P** | **C-index** |
| **SUVpeak** | **47** | **8.37** | **0.0036** | **6.15** | **1.47–25.6** | **0.013** | **0.626** |
| **SUVmax** | 43 | 16.9 | 0.033 | 2.63 | 1.02–6.78 | 0.045 | 0.579 |
| **SUVmean** | 39 | 9.00 | 0.026 | 2.40 | 1.05–5.48 | 0.038 | 0.579 |
| **SUVmin** | 39 | 4.06 | 0.00075 | 4.07 | 1.68–9.85 | 0.002 | 0.671 |
| **MTV** | 35 | 39.7 | 0.085 | 1.79 | 0.88–3.65 | 0.108 | 0.567 |
| **TLG** | 43 | 3021 | 0.00048 | 3.40 | 1.69–6.84 | <0.001 | 0.643 |
| **Baseline PET – Overall survival 12 months – ROC Cut-Off** | | | | | | | |
| **Parameter** | **n = 58** | **Cut-off** | **Log-rank P** | **HR** | **95%CI** | **P** | **C-index** |
| **SUVpeak** | 37 | 13.8 | 0.5 | 1.31 | 0.602–2.86 | 0.494 | 0.548 |
| **SUVmax** | 21 | 27.3 | 0.044 | 2.18 | 1.01–4.72 | 0.047 | 0.601 |
| **SUVmean** | 24 | 11.1 | 0.28 | 0.656 | 0.304–1.42 | 0.283 | 0.564 |
| **SUVmin** | 29 | 7.50 | 0.17 | 1.69 | 0.794–3.61 | 0.173 | 0.589 |
| **MTV** | **28** | **57.2** | **0.033** | **2.31** | **1.05–5.11** | **0.038** | **0.617** |
| **TLG** | 26 | 1005 | 0.059 | 2.09 | 0.958–4.58 | 0.064 | 0.603 |

PET = Positron-Emission-Tomography; PFS = Progression-free survival; OS = Overall survival; AUC = Area under the curve; HR = Hazard-Ratio; ROC = Receiver operating characteristics; Cox = Cox proportional regression analysis;

**Table 8S:** Comparison of ROC- and MSLRS- based cut-off analysis for Δ-parameters of first PET after BsAb initiation

| **PET-Parameter / PFS** | **AUC** | **AUC P** | **ROC Cut-Off** | **KM P** | **HR** | **Cox P** | **MSLRS Cut-Off** | **KM P** | **HR** | **Cox P** |
| --- | --- | --- | --- | --- | --- | --- | --- | --- | --- | --- |
| SUVmax | 0.75 | 0.006 | >-0.57 | 0.003 | 4.23 | 0.01 | >-0.785 | 0.0021 | 5.47 | 0.0068 |
| SUVpeak | 0.8 | 0.001 | >-0.45 | 0.005 | 3.53 | 0.014 | >-0.782 | 0.0024 | 6.89 | 0.0096 |
| SUVmean | 0.75 | 0.005 | >-0.75 | 0.003 | 5.11 | 0.009 | >-0.755 | 0.0011 | 7.65 | 0.0065 |
| SUVmin | 0.74 | 0.009 | >-0.56 | 0.002 | 5.28 | 0.008 | >-0.591 | 0.0015 | 5.62 | 0.0059 |
| whole-body MTV | 0.7 | 0.017 | >-0.49 | 0.102 | 2.06 | 0.136 | >1.64 | 0.0029 | 4.26 | 0.0054 |
| whole-body TLG | 0.73 | 0.007 | >-0.55 | 0.012 | 2.96 | 0.025 | >-0.861 | 0.0098 | 3.41 | 0.0171 |
| **PET-Parameter / OS** | **AUC** | **AUC**  **P** | **ROC**  **Cut-Off** | **KM P** | **HR** | **Cox P** | **MSLRS Cut-Off** | **KM P** | **HR** | **Cox P** |
| SUVmax | 0.78 | 0.001 | >-0.14 | 0.003 | 4.28 | 0.006 | >-0.141 | 0.0026 | 4.38 | 0.00574 |
| SUVpeak | 0.83 | 0.001 | >-0.45 | 0.01 | 4.41 | 0.02 | >-0.378 | 0.0069 | 4.19 | 0.0127 |
| SUVmean | 0.77 | 0.001 | >-0.21 | 0.01 | 3.6 | 0.017 | >-0.755 | 0.0038 | 11.1 | 0.0013 |
| SUVmin | 0.72 | 0.011 | >-0.42 | 0.003 | 6.78 | 0.01 | >-0.545 | 0.0031 | 6.89 | 0.0107 |
| whole-body MTV | 0.73 | 0.008 | >-0.49 | 0.063 | 3.04 | 0.08 | >0.126 | 0.016 | 3.35 | 0.0228 |
| whole-body TLG | 0.75 | 0.003 | >-0.55 | 0.005 | 4.86 | 0.013 | >-0.779 | 0.0055 | 4.93 | 0.0124 |

PET = Positron-Emission-Tomography; PFS = Progression-free survival; AUC = Area under the curve; ROC = Receiver operating characteristics; KM = Kaplan-Meier; HR = Hazard-Ratio; Cox (Cox proportional regression analysis); MSLRS = maximally selected long-rank statistical analysis;

**Table 9S:** ROC-AUC analysis of PET parameters from the first PET after BsAb initiation to predict the progression-free survival after 6 months

| **Parameter** | **N** | **Events** | **AUC** | **95% CI** | **p-value** | **Cut-off** | **Sensitivity** | **Specificity** | **Youden J** |
| --- | --- | --- | --- | --- | --- | --- | --- | --- | --- |
| SUVpeak | 38 | 22 | 0.722 | 0.551–0.893 | 0.060 | −0.852 | 0.955 | 0.438 | 0.392 |
| SUVmean | 38 | 22 | 0.685 | 0.499–0.871 | 0.123 | −0.768 | 0.864 | 0.562 | 0.426 |
| SUVmin | 38 | 22 | 0.685 | 0.495–0.875 | 0.138 | −0.284 | 0.727 | 0.688 | 0.415 |
| SUVmax | 38 | 22 | 0.682 | 0.494–0.870 | 0.130 | −0.304 | 0.682 | 0.688 | 0.369 |
| TLG | 38 | 22 | 0.602 | 0.418–0.787 | 0.314 | −0.253 | 0.545 | 0.688 | 0.233 |
| MTV | 38 | 22 | 0.563 | 0.376–0.749 | 0.470 | 1.74 | 0.273 | 1.000 | 0.273 |

PET = Positron-Emission-Tomography; PFS = Progression-free survival; AUC = Area under the curve; ROC = Receiver operating characteristics; Cox = Cox proportional regression analysis;

**Table 10S:** ROC-AUC analysis of PET parameters from the first PET after BsAb initiation to predict the overall survival after 12 months

| **Parameter** | **N** | **Events** | **AUC** | **95% CI** | **p-value** | **Cut-off** | **Sensitivity** | **Specificity** | **Youden J** |
| --- | --- | --- | --- | --- | --- | --- | --- | --- | --- |
| SUVmin | 38 | 30 | 0.579 | 0.351–0.808 | 0.307 | −0.154 | 0.533 | 0.750 | 0.283 |
| SUVmean | 38 | 30 | 0.562 | 0.312–0.813 | 0.304 | −0.818 | 0.800 | 0.500 | 0.300 |
| MTV | 38 | 30 | 0.525 | 0.333–0.717 | 0.159 | −0.247 | 0.567 | 0.750 | 0.317 |
| SUVmax | 38 | 30 | 0.517 | 0.288–0.746 | 0.156 | −0.157 | 0.467 | 0.750 | 0.217 |
| SUVpeak | 38 | 30 | 0.513 | 0.267–0.758 | 0.188 | −0.729 | 0.667 | 0.500 | 0.167 |
| TLG | 38 | 30 | 0.500 | 0.310–0.690 | 0.068 | 1.04 | 0.267 | 1.000 | 0.267 |

PET = Positron-Emission-Tomography; OS = Overall survival; AUC = Area under the curve; ROC = Receiver operating characteristics; Cox = Cox proportional regression analysis;

**Table 11S:** Kaplan-Meier survival analysis, Cox-Regression and C-Index evaluation for PET parameters from the first PET after BsAb initation predicting progression-free survival and overall survival after 6 and 12 months

| **First PET after BsAb initiation – Progression-free survival 6 months – ROC Cut-Off** | | | | | | | |
| --- | --- | --- | --- | --- | --- | --- | --- |
|  | | | | | | | |
| **Parameter** | **n = 38**  **High-Risk** | **Cut-off** | **Log-rank P** | **HR** | **95%CI** | **P** | **C-index** |
| **SUVpeak** | **30** | **−0.852** | **0.014** | **7.93** | **1.06–59.3** | **0.044** | **0.624** |
| **SUVmax** | 20 | −0.304 | 0.0083 | 3.36 | 1.28–8.81 | 0.014 | 0.645 |
| **SUVmean** | 26 | −0.768 | 0.0028 | 6.68 | 1.54–28.9 | 0.011 | 0.651 |
| **SUVmin** | 21 | −0.284 | 0.0052 | 3.81 | 1.37–10.6 | 0.011 | 3.81 |
| **MTV** | 6 | 1.74 | 0.0029 | 4.26 | 1.53–11.8 | 0.005 | 0.606 |
| **TLG** | 17 | −0.253 | 0.029 | 2.55 | 1.05–6.21 | 0.039 | 0.612 |
| **First PET after BsAb initiation – Overall survival 12 months – ROC Cut-Off** | | | | | | | |
| **Parameter** | **n = 38** | **Cut-off** | **Log-rank P** | **HR** | **95%CI** | **P** | **C-index** |
| **SUVpeak** | 24 | −0.729 | 0.014 | 5.29 | 1.19–23.4 | 0.028 | 0.618 |
| **SUVmax** | 16 | −0.157 | 0.0074 | 3.80 | 1.33–10.8 | 0.013 | 0.663 |
| **SUVmean** | **28** | **−0.818** | **0.015** | **8.46** | **1.09–65.6** | **0.041** | **0.609** |
| **SUVmin** | 18 | −0.154 | 0.017 | 3.37 | 1.18–9.65 | 0.024 | 0.643 |
| **MTV** | 19 | −0.247 | 0.27 | 1.78 | 0.622–5.12 | 0.282 | 0.558 |
| **TLG** | 8 | 1.04 | 0.055 | 2.66 | 0.938–7.52 | 0.066 | 0.587 |

PET = Positron-Emission-Tomography; PFS = Progression-free survival; OS = Overall survival; AUC = Area under the curve; HR = Hazard-Ratio; ROC = Receiver operating characteristics; Cox = Cox proportional regression analysis;

**Table 12S:** Univariate and multivariate Cox regression analysis for progression-free survival for clinical risk factors and baseline PET parameters

| **Variable** | **Univariate HR**  **(95% CI)** | **p-value** | **Multivariate HR**  **(95% CI)** | **p-value** |
| --- | --- | --- | --- | --- |
| Age* | 1.01 (0.66–1.54) | 0.977 | — | — |
| Sex (male) | 0.98 (0.48–2.00) | 0.963 | — | — |
| Pretreatments | 0.97 (0.77–1.23) | 0.805 | — | — |
| Ann Arbor II | 1.71 (0.31–9.34) | 0.537 | — | — |
| Ann Arbor III | 1.65 (0.32–8.50) | 0.552 | — | — |
| Ann Arbor IV | 2.10 (0.49–9.01) | 0.320 | — | — |
| Bulky disease | 3.03 (1.14–8.05) | 0.026 | 1.41 (0.44–4.52) | 0.562 |
| Extranodal disease | 1.84 (0.92–3.67) | 0.085 | 1.04 (0.43–2.53) | 0.927 |
| LDH* | 1.46 (1.12–1.89) | 0.005 | 1.12 (0.74–1.69) | 0.592 |
| ECOG 1 | 2.12 (0.83–5.41) | 0.115 | 1.70 (0.64–4.48) | 0.287 |
| ECOG 2 | 11.51 (3.14–42.25) | <0.001 | 4.23 (0.83–21.58) | 0.083 |
| ECOG 3 | 6.16 (1.20–31.59) | 0.029 | 4.67 (0.66–33.16) | 0.123 |
| IPI 1 | N/A | 0.998 | — | — |
| IPI 2 | N/A | 0.998 | — | — |
| IPI 3 | N/A | 0.998 | — | — |
| IPI 4 | N/A | 0.998 | — | — |
| IPI 5 | N/A | 0.998 | — | — |
| Baseline SUVmax* | 1.38 (0.83–2.31) | 0.214 | — | — |
| **Baseline whole-body MTV*** | 1.34 (1.12–1.59) | 0.001 | **1.55 (1.05–2.29)** | **0.028** |

*IQR-adjusted

HR = Hazard-Ratio;

**Table 13S:** Univariate and multivariate Cox regression analysis for overall survival for clinical risk factors and baseline PET parameters

| **Variable** | **Univariate HR**  **(95% CI)** | **p-value** | **Multivariate HR**  **(95% CI)** | **p-value** |
| --- | --- | --- | --- | --- |
| Age* | 1.10 (0.66–1.82) | 0.719 | — | — |
| Sex (male) | 0.82 (0.36–1.87) | 0.642 | — | — |
| Pretreatments | 0.99 (0.77–1.27) | 0.931 | — | — |
| Ann Arbor II | 0.57 (0.08–4.09) | 0.578 | — | — |
| Ann Arbor III | 1.26 (0.24–6.52) | 0.786 | — | — |
| Ann Arbor IV | 1.38 (0.31–6.07) | 0.674 | — | — |
| Bulky disease | 4.15 (1.53–11.24) | 0.005 | 2.68 (0.70–10.27) | 0.150 |
| Extranodal disease | 2.43 (1.09–5.42) | 0.030 | 2.16 (0.84–5.58) | 0.111 |
| LDH* | 1.66 (1.24–2.23) | <0.001 | 1.07 (0.63–1.80) | 0.813 |
| ECOG 1 | 2.87 (0.92–8.98) | 0.070 | 2.60 (0.78–8.70) | 0.120 |
| ECOG 2 | 6.54 (1.57–27.34) | 0.010 | **6.89 (1.24–38.18)** | **0.027** |
| ECOG 3 | 12.88 (2.18–76.17) | 0.005 | 9.15 (0.67–124.94) | 0.097 |
| IPI 1 | N/A | 0.999 | — | — |
| IPI 2 | N/A | 0.999 | — | — |
| IPI 3 | N/A | 0.998 | — | — |
| IPI 4 | N/A | 0.998 | — | — |
| IPI 5 | N/A | 0.998 | — | — |
| Baseline SUVmax* | 1.42 (0.83–2.42) | 0.197 | — | — |
| **Baseline whole-body MTV*** | 1.34 (1.10–1.63) | 0.003 | **1.82 (1.16–2.87)** | **0.010** |

*IQR-adjusted

HR = Hazard-Ratio;

**Table 14S:** Baseline PET parameters for prediction of overall survival using overall survival -derived cutoff

| **Overall survival** | | | | | | | |
| --- | --- | --- | --- | --- | --- | --- | --- |
| **Parameter** | **n = 58** | **Cut-off** | **Log-rank P** | **HR** | **95%CI** | **P** | **C-index** |
| SUVpeak | 47 (81%) | >7.9 | 0.026 | 4.72 | 1.07 – 20.87 | **0.0406** | 0.95 |
| SUVmax | 50 (86%) | >9.62 | 0.02 | N/A | N/A | N/A | N/A |
| SUVmean | 50 (86%) | >5.52 | 0.02 | N/A | N/A | N/A | N/A |
| SUVmin | 39 (67%) | >4.04 | 0.0028 | 5.1 | 1.54 – 16.89 | **0.0077** | 0.84 |
| whole-body MTV | 35 (60%) | >39.1 | 0.021 | 2.52 | 1.12 – 5.68 | **0.0259** | 0.76 |
| whole-body TLG | 20 (34%) | >1508.5 | 0.0043 | 2.98 | 1.36 – 6.51 | **0.00062** | 0.79 |

HR = Hazard-Ratio;

**Table 15S:** Baseline PET parameters for prediction of overall survival using progression-free survival-derived cutoff

| **Parameter** | **Log-rank P** | **HR** | **95% CI** | **Cox P** | **C-index** |
| --- | --- | --- | --- | --- | --- |
| MTV | 0.069 | 2.62 | 0.90–7.64 | 0.077 | 0.580 |
| TLG | 0.008 | 2.91 | 1.29–6.58 | 0.010 | 0.627 |

HR = Hazard-Ratio;

**Table 16S:** Parameters of first PET after BsAb initiation for overall survival prediction, using overall survival-derived cutoff

| **Overall survival** | | | | | | | |
| --- | --- | --- | --- | --- | --- | --- | --- |
| **Parameter** | **n** | **Cut-off** | **Log-rank P** | **HR** | **95%CI** | **P** | **C-index** |
| DS4-5 | 29 (76%) | N/A | 0.028 | 7.07 | 0.93 – 53.85 | 0.059 | 0.72 |
| ΔSUVpeak | 18 (47%) | >-0.378 | 0.0068 | 4.19 | 1.36 – 12.93 | **0.0127** | 0.8 |
| ΔSUVmax | 15 (40%) | >-0.141 | 0.0026 | 4.38 | 1.54 – 12.5 | **0.00574** | 0.81 |
| ΔSUVmean | 25 (66%) | >-0.755 | 0.0038 | 11.1 | 1.45 – 84.69 | **0.0013** | 0.95 |
| ΔSUVmin | 22 (58%) | >-0.545 | 0.0031 | 6.89 | 1.56 – 30.36 | **0.0107** | 0.85 |
| Δwhole-body MTV | 13 (34%) | >0.126 | 0.016 | 3.35 | 1.18 – 9.48 | **0.0228** | 0.85 |
| Δwhole-body TLG | 19 (50%) | >-0.779 | 0.0055 | 4.93 | 1.41 – 17.25 | **0.0124** | 0.79 |

HR = Hazard-Ratio;

**Table 17S:** Parameters of first PET after BsAb initiation for overall survival prediction, using progression-free survival-derived cutoff

| **Parameter** | **Log-rank P** | **HR** | **95% CI** | **Cox P** | **C-index** |
| --- | --- | --- | --- | --- | --- |
| SUVpeak | 0.018 | 7.84 | 1.04–59.15 | 0.046 | 0.630 |
| SUVmax | 0.014 | 5.29 | 1.19–23.44 | 0.028 | 0.618 |
| SUVmean | 0.004 | 11.10 | 1.45–84.69 | 0.020 | 0.654 |
| SUVmin | 0.009 | 5.81 | 1.31–25.66 | 0.020 | 0.649 |
| MTV | 0.062 | 2.74 | 0.91–8.22 | 0.073 | 0.592 |
| TLG | 0.059 | 3.10 | 0.89–10.82 | 0.075 | 0.611 |

HR = Hazard-Ratio;

**Figure 1S:** Comprehensive overview of image analysis of one case example

**
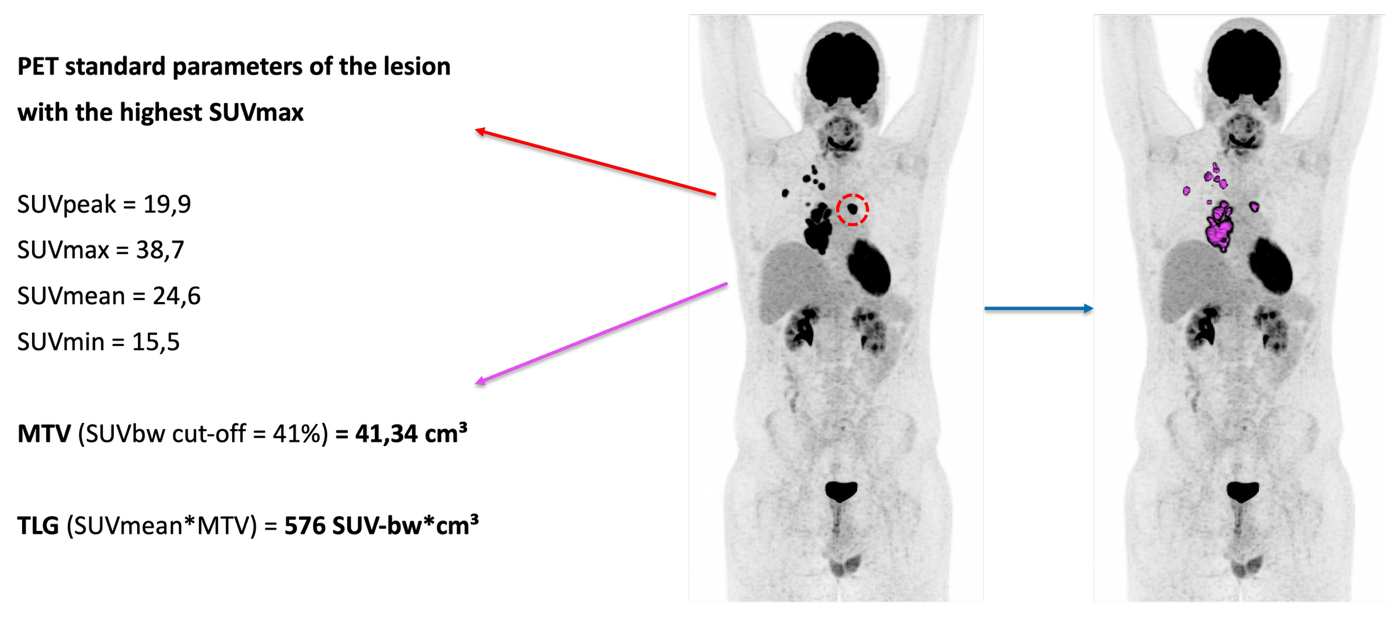
**

**Figure 2S:** Survival of the total cohort


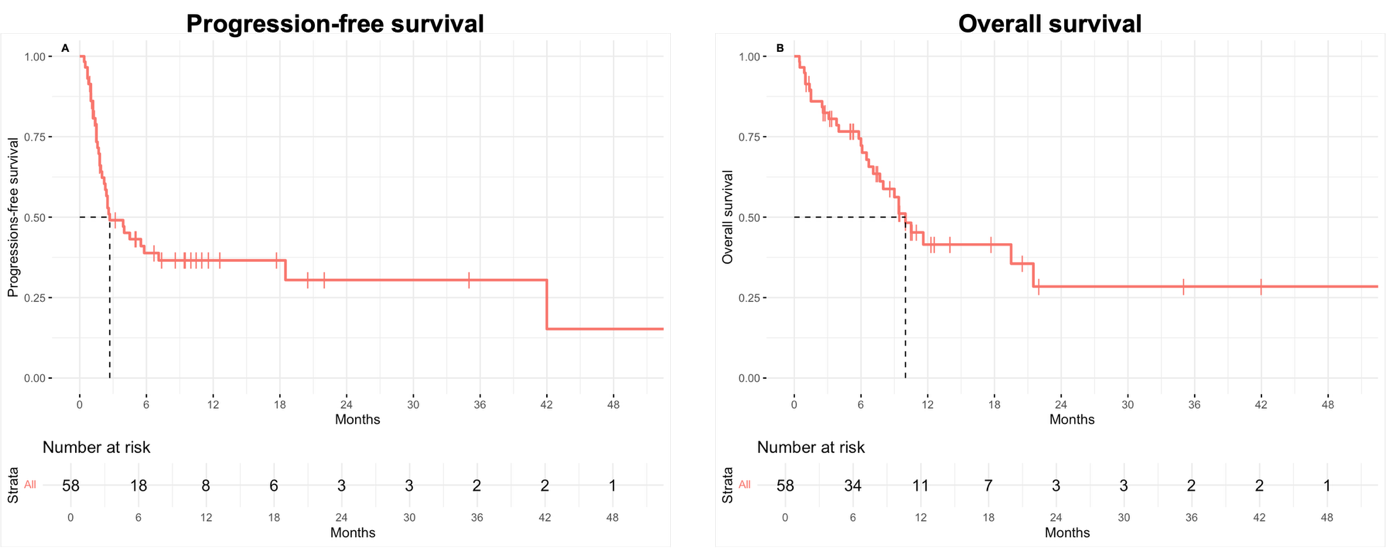


**Figure 2S:** In this multicenter cohort the median PFS was 2.7 months with 36.6% (21/58) of patients without progression at 12 months (A). The median OS was 10 months with 41.5% (24/58) of patients being alive at 12 months (B).

**Figure 3S:** Scanner-related variability of PET parameters in baseline PET and first PET after BsAb initiation


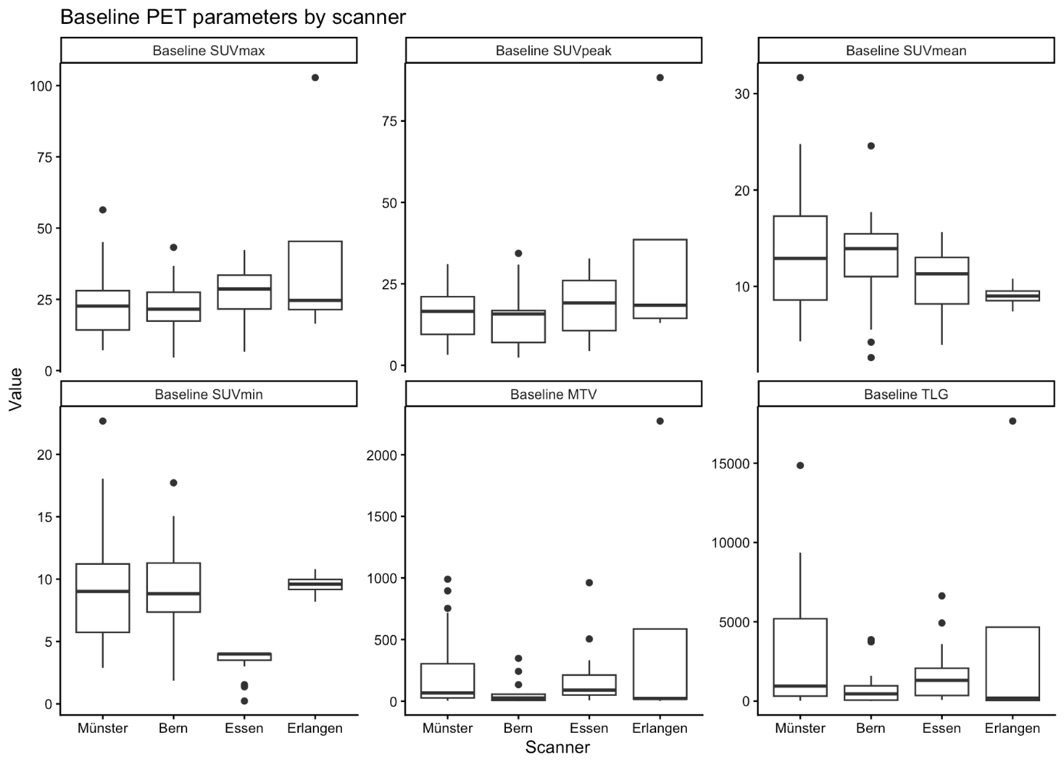


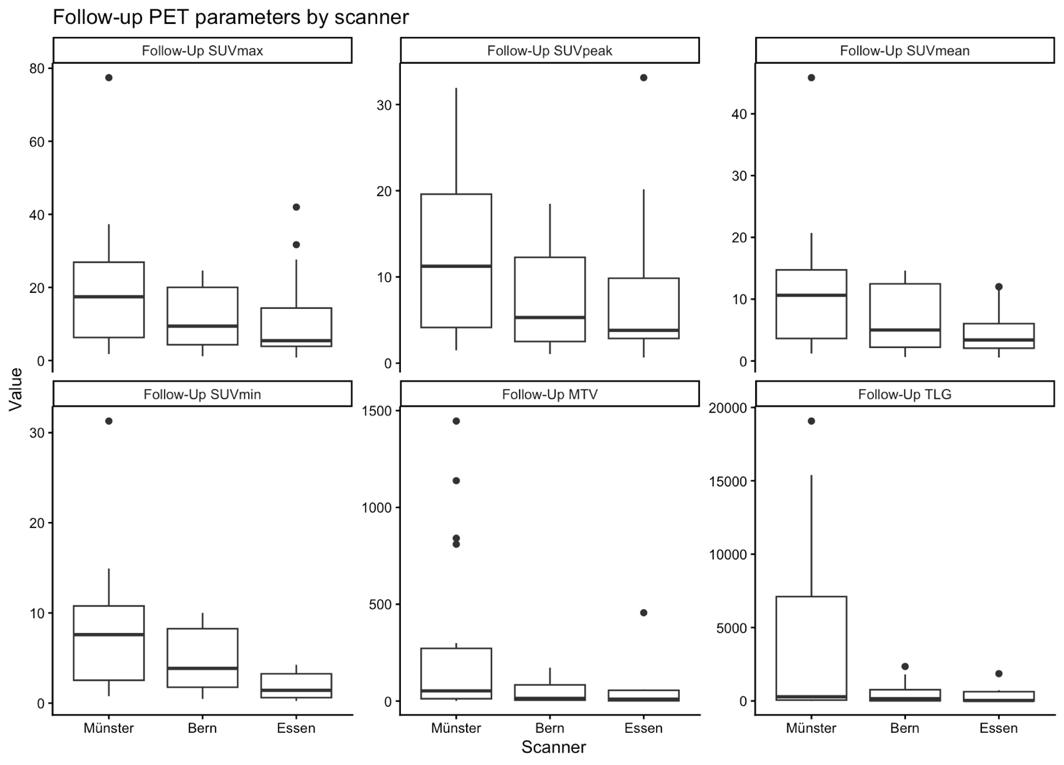


**Figure 4S:** Kaplan-Meier analysis of binarized ideal cut-off PET parameters (SUVmax, SUVmean, SUVmin, TLG) in the baseline PET. Patients are separated in a high-risk and low-risk group towards shorter PFS and OS. **
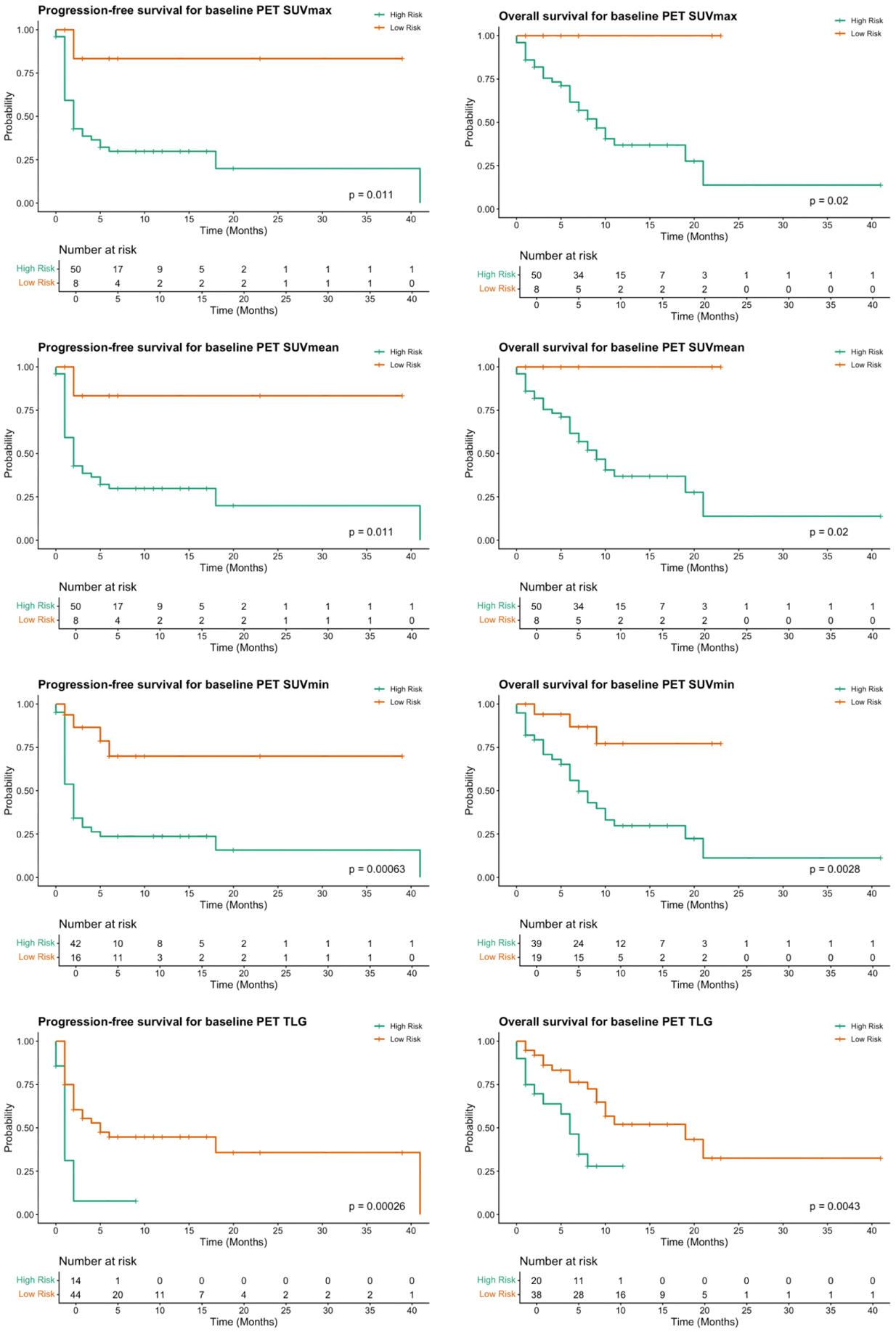
**

**Figure 5S:** PFS and OS analysis for ideal cut-off analysis of remaining ΔPET parameters of the first follow-up PET after BsAb initiation. Patients are separated in a high-risk and low-risk group towards shorter PFS and OS.


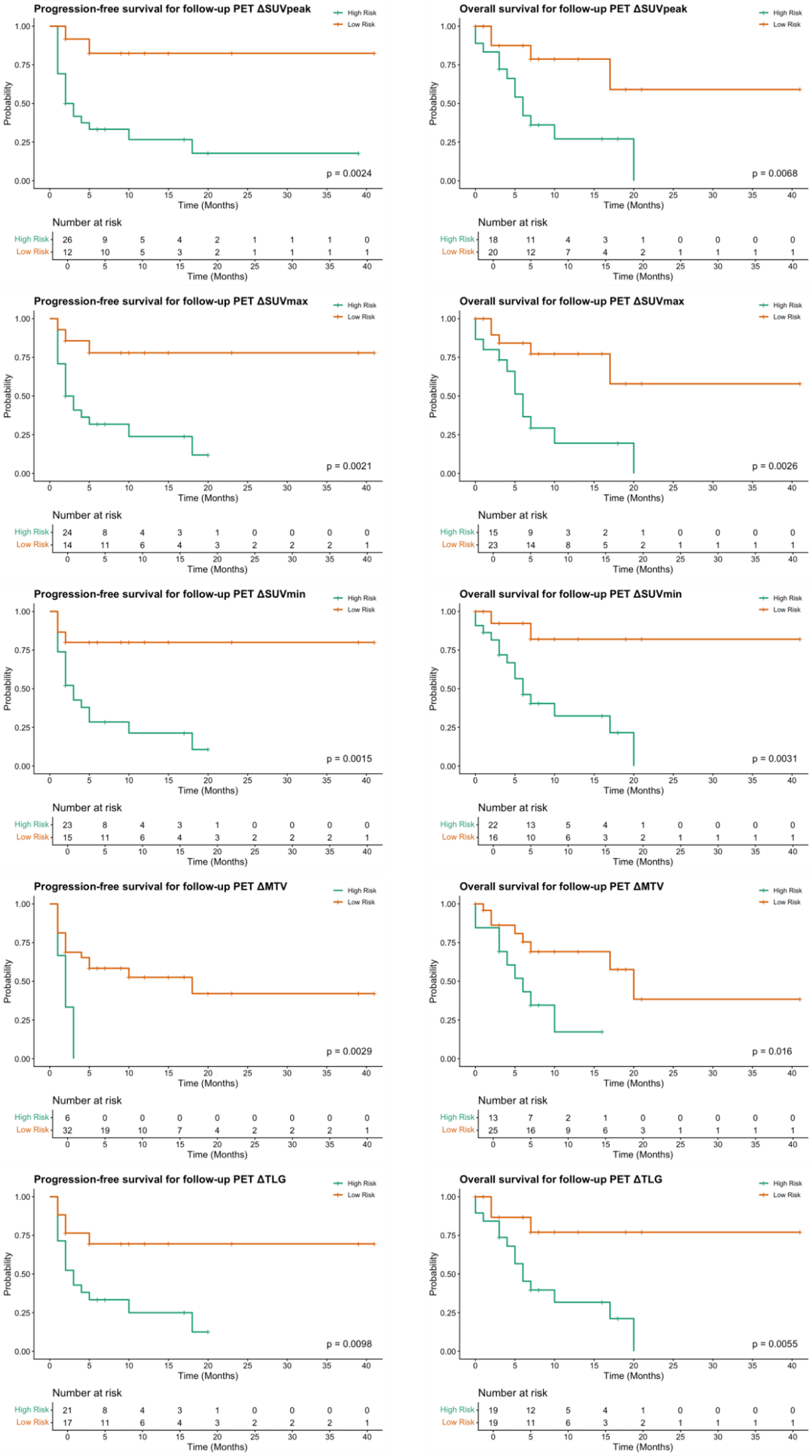


**Figure 6S:** Kaplan-Meier analysis of progression-free survival based on the predefined risk model (bulky disease, extranodal involvement, LDH, ECOG, whole body MTV). Patients are separated into a high-risk and low-risk group.


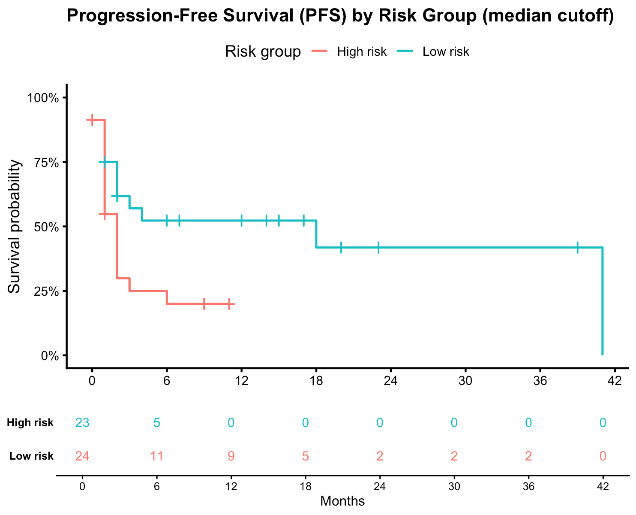


Cut-Off PFS: 0.6319, Log Rank P < 0.001, HR = 2.38 (1.11 – 5.1), P = 0.026

**Figure 7S:** Kaplan-Meier analysis of overall survival based on the predefined risk model (bulky disease, extranodal involvement, LDH, ECOG, whole body MTV). Patients are separated into a high-risk and low-risk group.


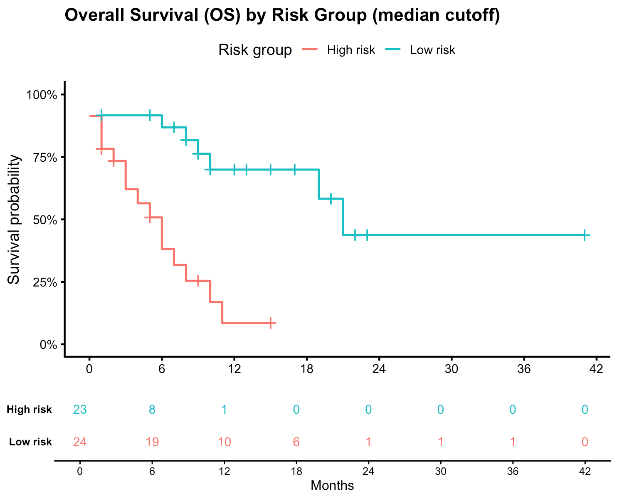


Cut-Off OS: 0.4126, Log Rank P < 0.001, HR = 5.83 (2.22 – 15.27), P < 0.001
